# Identification of Potential Migrants in Polyethylene Terephthalate Samples of Ecuadorian Market

**DOI:** 10.3390/polym13213769

**Published:** 2021-10-31

**Authors:** Karina Marín-Morocho, Sandra Domenek, Rómulo Salazar

**Affiliations:** 1 Escuela Superior Politécnica del Litoral, ESPOL, Facultad de Ingeniería en Mecánica y Ciencias de la Producción, Carrera de Ingeniería en Alimentos, Campus Gustavo Galindo, Km 30.5 Vía Perimetral, Guayaquil P.O. Box 09-01-5863, Ecuador; kamarin@espol.edu.ec; 2 UMR Ingénierie Procédés Aliments, AgroParisTech, INRA, Université Paris-Saclay, F-91300 Massy, France; sandra.domenek@agroparistech.fr

**Keywords:** polyethylene terephthalate, PET, chemicals compounds, migration, additives, bottles

## Abstract

Polyethylene terephthalate (PET) is the plastic packaging material most widely used to produce bottles intended for contact with food and beverages. However, PET is not inert, and therefore, some chemical compounds present in PET could migrate to food or beverages in contact, leading to safety issues. To evaluate the safety of PET samples, the identification of potential migrants is required. In this work, eight PET samples obtained from the Ecuadorian market at different phases of processing were studied using a well-known methodology based on a solvent extraction followed by gas chromatography–mass spectrometry analysis and overall migration test. Several chemical compounds were identified and categorized as lubricants (carboxylic acids with chain length of C12 to C18), plasticizers (triethyl phosphate, diethyl phthalate), thermal degradation products (p-xylene, benzaldehyde, benzoic acid), antioxidant degradation products (from Irgafos 168 and Irganox), and recycling indicator compounds (limonene, benzophenone, alkanes, and aldehydes). Additionally, overall migration experiments were performed in PET bottles, resulting in values lower than the overall migration limit (10 mg/dm^2^); however, the presence of some compounds identified in the samples could be related to contamination during manufacturing or to the use of recycled PET-contaminated flakes. In this context, the results obtained in this study could be of great significance to the safety evaluation of PET samples in Ecuador and would allow analyzing the PET recycling processes and avoiding contamination by PET flakes from nonfood containers.

## 1. Introduction

Polyethylene terephthalate (PET) is a plastic packaging material commonly used to produce bottles for drinking water, mineral water, carbonated beverages, and edible oils [1]. This semicrystalline polyester is an excellent packaging material due mainly to its good gas barrier properties, good thermal and mechanical properties, light weight, transparency, strength, good processability, and good recyclability [2]. Moreover, PET can be considered as a high-inertness material and a low-additive packaging [3], which leads to a low mass transfer from PET packaging to foodstuff in contact and limited issues of quality and safety [4].

PET bottle production generally consists of two phases: injection and blow-molding. In the injection phase, PET pellets are used to obtain amorphous preforms. Then, in the blow-molding phase, preforms are stretched to obtain biaxially oriented bottles. Some substances can be added to PET to improve the performance during the processing of bottles and to enhance their functional properties. Therefore, these additives could migrate to food in contact. Additionally, the bottle manufacturing processes can lead to polymer and additive degradation, generating compounds that could be potential migrants from PET bottles into foodstuff in contact [5].

In the case of plastic packaging materials, migration tests for checking compliance are required in all articles intended to come into contact with foodstuffs, according to EU regulations [6,7,8], where the use of simulants under specific conditions of temperature and storage time is regulated. The overall migration is related to the inertness of a plastic material. It is defined as the mass of material transferred to a food simulant, which is determined by a standardized test. The overall migration limit is the maximum permitted amount of nonvolatile substances that a plastic article can release into food simulants. However, migration tests are expensive and time-consuming, and they are usually complex due mainly to the low concentration of migrant compounds in food simulants [3], leading to identification and quantification issues. Consequently, comprehensive migration studies frequently start with analyzing the polymer or its extract in solvents to identify the potential migrants before contact with foodstuff [3,9,10,11,12].

The methodologies to identify the potential migrants in plastic packaging materials commonly use solvent extraction followed by chromatographic separations and mass spectrometric detections, approaches which are complex and require laborious sample preparation [4,13,14,15,16]. Confident identification of potential migrants can be carried out by gas chromatography coupled with mass spectrometry detection (GC-MS) using mass spectral matches with a database and Kovats retention index [11]. For a given GC stationary phase, the Kovats retention index of a compound is a characteristic value obtained by interpolation, relating the adjusted retention time of the molecule to the adjusted retention time of two alkanes eluted before and after the peak of the sample component.

The migration of chemical compounds from PET bottles to a medium in contact has been extensively reported in the literature [17,18,19,20]. Several types of additives have been identified in PET bottles intended for food contact. Thus, plasticizers such as di-2-ethylhexylphthalate (DEHP), dioctyl phthalate (DOP), and dibutyl phthalate (DBP) were reported in PET bottles intended for beverages and spring water [21,22]. Additionally, DEHP was identified in PET bottles intended for contact with yogurt [23]. Moreover, literature reported the presence of alkanes (tetradecane, eicosane), antioxidants (Irganox 1076 and phenol, 2,4-bis(1,1-dimethylethyl)-), and plasticizers (diisooctyl phthalate, (DIOP)) in soda and water PET bottles or juice packages [24]. The presence of carboxylic acids and related compounds have been reported in PET soda and water bottles, such as octadecanoic acid and octadecanoic acid, 2-hydroxy 1-(hydroxymethyl) ethyl ester [24]. Other kinds of contaminants in PET bottles are related to the use of PET flakes from postconsumer recycled PET (rPET), such as flavor compounds, degradation products from the polymer, and chemical products from misused PET bottles [2,3].

In 2013, Ecuador implemented a regulatory framework for plastic materials and articles intended for food contact, adopting the European regulation about overall and specific migration. In the case of PET, the recycling chain to produce rPET in this market is relatively recent compared with developed countries. Moreover, to our best knowledge, no literature has been reported about the chemical compounds present in PET bottles in Ecuador. Thus, the objective of this work was to identify the potential migrants in PET samples at different phases of processing, such as resins, preforms, and bottles obtained from various suppliers of the Ecuadorian market. Samples were analyzed by GC-MS, and the compounds identified were then categorized according to their use and application in plastic packaging. Additionally, the overall migration of PET bottles was determined with the aim of evaluating the compliance of bottles with the overall migration limit established by regulations.

## 2. Materials and Methods

### 2.1. Materials

#### 2.1.1. Chemicals and Reagents

Chloroform (99.5%), ethanol (99.9%), and a standard solution of a series of alkanes (C7-C40, 1000 μg/mL of each component in hexane) were obtained from Sigma Aldrich (Saint Louis, MO, USA).

#### 2.1.2. PET Samples

Samples of virgin PET pellets, preforms, and bottles were supplied by Ecuadorian companies specialized in the manufacturing of PET preforms and bottles intended for contact with beverages, mineral water, and edible oil.

Two batches of samples were collected, each consisting of virgin pellets, preforms, and bottles. Additionally, 2 types of bottles were collected, resulting in the obtention of a total of 8 samples that were studied in this work. Batches were labeled P and T and bottles were labeled A and D, in reference to the name of the supplier company.

The preform samples were ground using a Retsch MM 400 universal laboratory mill (Haan, Germany) in presence of liquid nitrogen to prevent any degradation during grinding; grinding was performed in order to increase the surface area and thus to improve the extraction efficiency. In the case of bottles, samples were cut into small square pieces of around 5 mm per side.

### 2.2. Methods

#### 2.2.1. Extraction of Chemical Compounds from PET Samples

For all samples, 5 g of material was placed in a cellulose thimble (Whatman International, Maidstone, UK) and put in a Soxhlet apparatus with 200 mL of chloroform (16 h, 62 °C) in order to extract the chemical compounds from PET samples. The speed of Soxhlet extraction was equivalent to one cycle every 20–30 min. The extraction of each sample was carried out in triplicate.

Then, extracts were concentrated to approximately 5 mL using a rotary evaporator (ROVA-100, London, UK) at 40 °C. After, the extract was filtered using a polytetrafluoroethylene (PTFE) membrane with a pore size of 0.45 µm (General Electric Scientific, Pittsburgh, PA, USA) and was stored in a freezer at 4 °C before being analyzed.

#### 2.2.2. Analysis of PET Samples by GC-MS 

A volume of 2 μL of sample concentrated extract was injected in splitless mode into a gas chromatograph (GC) from Agilent Technologies, Model 7890A, coupled with a mass spectrometer 5975 INSERT. The GC was equipped with a capillary column DB5-MS (Agilent J&W Scientific, 30 m length × 250 µm inner diameter × 0.25 µm film thickness). The carrier gas was He at 0.9 mL/min. The oven temperature program began with an initial temperature of 40 °C for 1 min, and then the temperature was increased at a rate of 3 °C/min to 300 °C and then maintained for 25 min. 

The mass spectrometer parameters used for identification of chemical compounds were as follows: electron impact ionization; electron energy, 15 eV; ion source, 230 °C; electron multiplier voltage, 3000 eV; transfer line, 305 °C; scanning, between 50 and 700 amu. The data were recorded by MSD ChemStation software (Agilent Technologies, Palo Alto, CA, USA) and the identification of the constituents was achieved using mass spectral matches with Wiley7 NIST 05 mass spectra database. To confirm identification, Kovats indices were determined. For that, a standard mixture of alkanes C7 to C40 in hexane was analyzed under the same conditions as the samples.

#### 2.2.3. Overall Migration Test

The overall migration (OM) was measured using a gravimetric method, in which the sample residue was weighed after the evaporation of a food simulant. The test conditions and simulant used for the OM assays were chosen according to the European Regulation 10/2011 [8]. In this work, the OM test was carried out at 40 °C for 10 days using the food simulant A (ethanol 10% *v/v*). PET bottle samples were cut into squares of 10 cm × 10 cm, and then they were cut into smaller pieces of 2.5 cm × 2.5 cm. PET squares were placed in a glass bottle with 100 mL of food simulant and sealed using a screw cap with silicon/PTFE septa. Then, the glass bottle was placed in an oven (Memmert, Schwabach, Germany) at 40 °C. Three replicates for each PET bottle sample were carried out. After 10 days of conditioning time, the bottles were removed from the oven, and the squares of PET samples were removed from the bottles. Then, the bottles containing the simulant were placed in an oven at 115 °C in order to evaporate the simulant. When the bottles were dried, they were put inside a glass desiccator to reach room temperature. Finally, the bottles were weighed by an analytical balance (Sartorious, Göttingen, Germany, 10^−4^ g) to verify that they reached constant weight. Finally, overall migration was determined by applying Equation (1):

(1) $$M=\frac{m_{2}-m_{1}}{S}\times 1000$$

where *M* is the overall migration in mg/dm^2^, *m*_1_ is the weight glass vial before overall migration, *m*_2_ is the weight glass vial after overall migration, and *S* is the area of PET in contact with food simulant.

## 3. Results and Discussion

With the purpose of identifying the potential migrants in PET samples, a methodology based on solvent extraction followed by GC-MS analysis was used. The identification of chemical compounds was carried out using mass spectral matches with the Wiley7 NIST 05 mass spectra database and confirmed comparing their Kovats indices.

**Figure 1:**
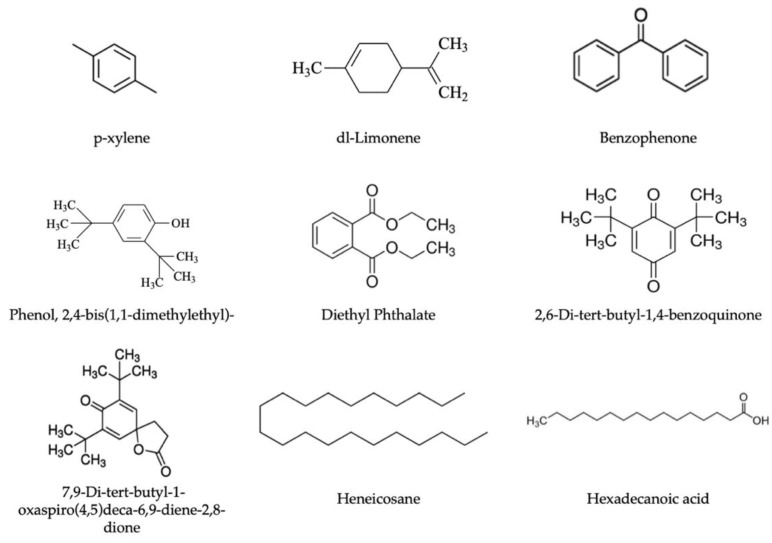
Structures of common compounds identified in PET samples.

The results showed the presence of several chemical compounds in the PET samples studied, identifying a total of 22 compounds; among them, 6 were identified in pellets, 9 were identified in preforms, and 20 were identified in bottles. The compounds identified in pellets and preforms are listed in Table 1, whereas those in PET bottles are presented in Table 2. Figure 1 presents some common compounds identified in the PET samples.

After identification, the compounds were categorized according to their use and application in plastic packaging, such as lubricants, plasticizers, products of thermal degradation, degradation products of antioxidants, and recycling indicators. The chemical compounds identified are discussed below by category.

### 3.1. Lubricants

Lubricants are a group of additives used to reduce the friction between the equipment surfaces and the polymer, promoting the flow of plastic over and through the equipment and preventing the plastic from sticking to the surfaces. The main lubricants used in plastic packaging materials are carboxylic acids, their esters and amides, polyethylene waxes, paraffin, stearates, and silicones. Generally, lubricants can be added to the plastic resin or applied externally [25].

Lubricants identified in this study were carboxylic acids with chain lengths varying from C12 to C18, which are shown in Table 1 and Table 2. Hexadecanoic acid and octadecanoic acid were found in all analyzed samples, which could indicate their extensive use as main components of lubricants in samples studied. The presence of hexadecanoic acid and octadecanoic acid in PET bottles was reported by literature [26], as was the presence of tetradecanoic acid, 1-methylethyl ester in PET pellets, preforms, and bottles [27]; however, to the best of our knowledge, there are no studies reporting the presence of dodecanoic acid, tetradecanoic acid, pentadecanoic acid, or heptadecanoic acid in PET samples. The carboxylic acids are generally recognized as safe (GRAS), and they are authorized by EU regulation to be used as additives or polymer production aids. 

### 3.2. Plasticizers 

Plasticizers are used in plastic packaging materials to improve their flexibility, extensibility, and processability. In this work, two plasticizers were found: triethyl phosphate and diethyl phthalate. The first one, triethyl phosphate, was identified in bottles P and D. The organophosphorus compounds are frequently used as plasticizers and flame retardants in plastic packaging; however, they are also used as antifoaming agents and additives in products such as lubricants and hydraulic fluids [28,29]. This compound has been reported as a plasticizer intended for food-contact plastics [30].

The second compound, diethyl phthalate, was found in pellets and in all bottles studied as shown in Table 1 and Table 2. According to the literature, phthalate esters are not used as plasticizers or additives in the manufacture of PET or PET bottles, nor they are used as substrates or precursors in the production of PET [2,5]. Contrary to this, several studies have identified these compounds in PET materials and PET bottled water [31,32]. Until now, the presence of phthalate esters in PET has not been satisfactorily explained, but their presence in very low amounts could be due to contamination during manufacturing or transport [2].

Some phthalates plasticizers for PET have been reported, such as dipentyl phthalate (DPP), diethyl phthalate (DEP), diisobutyl phthalate, and dibutyl phthalate (DBP) [31]. Bis(2-ethylhexyl) phthalate and di-octyladipate were identified by GC-MS in samples of PET bottles of juice and soft drink from the Australian market [22]. Moreover, di(2-ethylhexyl) phthalate and di(2-ethylhexyl) adipate were reported in PET samples from the Iranian market [23]. Additionally, the presence of diisobutyl phthalate and dibutyl phthalate was reported in PET bottles from the Lebanese market [27].

The PET bottle manufacturing process includes several steps where the polymer is heated to temperatures above its Tg, promoting the diffusion of contaminants within the polymer. The step of blowing is considered the most critical because the preforms are exposed to compressed air and high temperatures (>Tg) in order to ensure their biaxial orientation. So, one source of contamination of phthalates could be attributed to the compressed air that comes from pumps, filters, pipes, etc. [27]. On the other hand, the presence of phthalates in PET bottles could be explained by impurities in the starting materials due to the use of recycled PET-contaminated flakes [33]. 

### 3.3. Thermal Degradation Products 

Additionally, Table 1 and Table 2 show the presence in PET samples of p-xylene, benzaldehyde, and benzoic acid, which are thermal degradation products of PET [34]. Regarding p-xylene, it was found in preforms T and bottles P and D. The presence of this compound is attributed to thermal degradation of PET that generally occurs during manufacturing at a temperature between 200 and 300 °C [21]. Benzaldehyde was identified in preforms T whereas benzoic acid was found in preforms T and bottles T. These chemical compounds are also products of PET thermal degradation [35].

In fact, during PET manufacturing, several degradation and decomposition reactions can occur. High temperatures and the presence of oxygen in the PET can promote thermo-mechanical and thermo-oxidative reactions generating numerous compounds in the polymer. In this context, several studies about the PET thermal degradation process have been reported in the literature [34,35,36]. The main compounds found in PET bottles produced by thermal degradation were aldehydes (acetaldehyde, formaldehyde, and benzaldehyde), aliphatic hydrocarbons (C1–C4), aromatic hydrocarbons (benzene, toluene, ethylbenzene, xylene, and styrene), and esters (vinyl benzoate, methyl acetate) [21,37].

### 3.4. Degradation Products of Antioxidants 

In PET samples, three compounds were found that can be categorized as degradation products of antioxidants: 2,4-bis(1,1dimethyethyl) phenol in bottles P, T, and D; 2,6-di-tert-butyl-1,4-benzoquinone in bottles T and D; and 3,5-di-tert-butyl-4 hydroxybenzaldehyde in preforms P (Table 1 and Table 2). Literature has reported these products in PET samples [38,39]. The first two compounds are degradation products of polymer additives such as Irgafos 168 and Irganox 1010 used in plastic formulations [40,41], and the last compound is a degradation product of BHT (dibutylated hydroxytoluene) [21,27], which is commonly used as an antioxidant in plastic packaging materials and it is included in the positive list of additives to plastic materials intended for contact with foodstuff [8].

The presence of air during the melt processing of PET can lead to thermo-oxidative degradation. In order to prevent this degradation, antioxidants are added during polycondensation [42]. Between the additives, antioxidants are used to inhibit or retard the manifestations of aging or discoloration and the effects of oxidation on the chemical structure of the polymers [41].

Moreover, Table 2 shows in bottles A the compound identified as 7,9-di-tert-butyl-1-oxaspiro(4,5)deca-6,9-diene-2,8-dione, which is a degradation product of Irganox, according to the literature [43].

### 3.5. PET Recycling Indicators 

The results show the presence of two compounds that can be related to postconsumer recycled PET: limonene and benzophenone. Limonene was identified in preforms T and bottles T and D, whereas benzophenone was found in bottles P, T, and D (Table 1 and Table 2). These two molecules have been already reported in the literature on recycled PET flakes [17,19]. Limonene is typically found in postconsumer PET flakes, and it is related to prior contents of the recycled PET bottles, such as flavoring components. Regarding limonene, the literature reported the difficulty of cleaning this compound from the PET bottles because limonene can be sorbed in the PET polymer matrix [44]. Benzophenone is a ketone that can be added to plastic packaging as a UV blocker to protect against photo-degradation, and its use as an additive in plastic materials intended for contact with food is authorized with a specific migration limit of 0.6 mg/kg [8]. In the case of benzophenone, it was detected in PE/PET films from the Spanish market, and its presence could be linked to the printing inks used in the external surface of packaging films [45]. 

The bottle-to-bottle recycling process of PET must successfully reduce the concentrations of chemical compounds sorbed previously by the bottles in their first use [20]; however, the process of conventional washing in water of PET bottles is not capable of removing all the impurities from soft drink bottles and edible oil bottles [46]. According to literature, the main chemical compounds linked to recycled PET are limonene; aliphatic aldehydes; benzaldehyde; carboxylic acids (C8–C15); p-xylene; isopropyltoluene; cyclohexyl toluene; alkanes; plasticizers such as dibutyl phthalate(DBP), di(2-ethylhexyl)-adipate (DEHA), dioctyl phthalate (DOP), and diisononylphthalate (DINP); benzophenone; and alcohol groups (C12–C18) [17,19].

Additionally, two alkanes were identified in PET samples. Hexadecane was found in bottles P, and heneicosane was identified in bottles P, T, and A. The presence of alkanes was reported in the recycled PET [17], and hexadecane was found in PET postconsumer recycled flakes from nonfood containers [47]. So, alkanes found in the samples studied in this work could be related to the use of PET recycled flakes from nonfood containers in the production of new PET bottles intended for food contact.

Finally, two aliphatic aldehydes were identified in samples. Nonanal was found in bottles P, A, and D, whereas decanal was identified in bottles D. These compounds are very common in flavored soft drinks containers (Nerin 2003). In this sense, the presence of these aldehydes in PET bottles could be related to the use of recycled PET for the manufacture of bottles.

Here, it should be noted that in 2018 the industrial sector of beverages of Ecuador announced the use of around 25% of recycled material in the production of PET bottles, showing this value on the labels of bottles. Additionally, in December 2020, a new national regulation on single-use plastics products was adopted in Ecuador, giving a term of 4 years to the industrial sector to use 30% of recycled PET in the production of bottles [48]. Consequently, the analytical identification of chemical compounds from PET and recycled PET bottles is an important tool for the safety of the Ecuadorian market.

### 3.6. Chemical Compounds Identified at Different Phases of the Processing

In order to identify the chemical compounds at different phases of the processing of PET bottles, we compared the results obtained in pellets, preforms, and bottles of suppliers P and T. The results showed 13 chemical compounds identified in samples P and 12 compounds in samples T, as shown in Table 1 and Table 2. The common compounds present in pellets, preforms, and bottles were lubricants: dodecanoic, tetradecanoic, hexadecenoic, and octadecanoic acids in samples P and tetradecanoic, hexadecenoic, and octadecanoic acids in samples T. 

Additionally, compounds identified in preforms but not in pellets were 3,5-di-tert-butyl-4-hydroxybenzaldehyde in preforms P and p-xylene, benzaldehyde, dl-limonene, and benzoic acid in preforms T. As mentioned before, the presence of 3,5-di-tert-butyl-4-hydroxybenzaldehyde is related to the degradation of butylated hydroxytoluene (BHT) [49], whereas benzaldehyde and dl-limonene are recycling indicators, and p-xylene and benzoic acid are products formed from thermal degradation of the polymer. 

The compounds identified in preforms and bottles T were dl-limonene and benzoic acid, the first one being a recycling indicator and the second one being a product formed from thermal degradation of the polymer. 

Regarding the compounds identified only in PET bottles, the results showed the presence of p-xylene, nonanal, triethyl phosphate, phenol, 2,4-bis(1,1-dimethylethyl)-, and hexadecane in bottles P and 2,6-di-tert-butyl-1,4-benzoquinone, phenol, 2,4-bis(1,1-dimethylethyl)-, heptadecanoic acid, and heneicosane in bottles T. The presence of p-xylene, nonanal, 2,6-di-tert-butyl-1,4-benzoquinone, and phenol, 2,4-bis(1,1-dimethylethyl)- could be explained by thermal degradation of polymer or additives during the processing of bottles. Moreover, the compounds identified in PET bottles such as triethyl phosphate and heptadecanoic acid should show their use as plasticizers in PET. Finally, the results showed possible contamination of bottles P and T with hexadecane and heneicosane by using PET flakes from nonfood containers.

**Figure 2:**
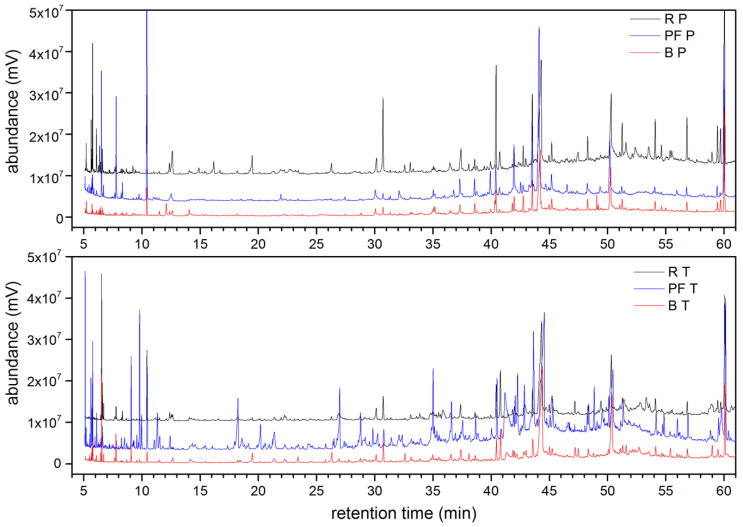
Chromatogram overlay of PET samples P (**Top**) and T (**Bottom**). R, PF, and B mean pellets, preforms, and bottles, respectively.

From a qualitative point of view, it can be mentioned that the chromatograms of the preform samples presented a higher number of peaks compared to the chromatograms of the bottle samples. The pellets presented the lowest number of peaks, as can be observed in Figure 2 (samples P and T). This could be attributed to the thermal processing of the samples, which increases the presence of volatile and semivolatile compounds from the degradation of PET and additives, as has been reported in the literature [21,35,40]. In P and T samples, a slight decrease in the numbers of peaks could be observed by shifting from preforms to bottles. This could be explained by the loss of these compounds by evaporation during the process and especially during the blowing [21].

Additionally, some peaks present in the chromatograms of PET samples could not be identified, due to the limits of the methodology used in this study; however, the main unknown peaks detected in the samples are presented as supplementary information.

### 3.7. Overall Migration Test

The overall migration tests of the PET bottles were carried out using food simulant “A” (aqueous foods), according to European regulation. The results of global migration were 1.2 ± 0.32 mg/dm^2^ in bottles P, 4.7 ± 1.44 mg/dm^2^ in bottles T, 3.00 ± 0.79 mg/dm^2^ in bottles A, and 3.40 ± 0.67 mg/dm^2^ in bottles D. These results were lower than the maximum limit established by regulations (10 mg/dm^2^) [8]; however, they are between 4 and 16 times higher than the values reported in the literature, where the overall migration of PET bottles into food simulant water was around 0.33 mg/dm^2^ [50].

## 4. Conclusions

Several potential migrants were identified in eight PET samples intended for contact with food: 6 compounds in resins, 9 compounds in preforms, and 20 compounds in PET bottles. The compounds identified were categorized as lubricants (C12–C18 carboxylic acids), plasticizers, products of thermal degradation, degradation products of antioxidants, and recycling indicators. Hexadecanoic acid and octadecanoic acid were found in all samples, which could be related to their use as lubricant components in the studied samples. Diethyl phthalate and alkanes found in the bottles studied in this work could be explained by contamination during processing or the use of PET recycled flakes from nonfood containers. Moreover, the values of overall migration found in PET bottle samples were lower than the limit established by regulations; consequently, they may be used for contact with water and soft drinks. The results obtained in this study could be of great significance to the safety evaluation of PET samples in Ecuador and would allow analyzing the PET recycling processes and avoiding contamination by PET flakes from nonfood applications. 

## Figures and Tables

**Table 1:** GC-MS peak relative area ^a^ of compounds identified in PET pellets and preforms P and T.

Tr (min)	Compounds	Function/Origin	Mw	Formula	CAS	KIcal ^b^	KI Ref ^c^	Pellets		Preforms	
								P	T	P	T
5.64	p-Xylene	Thermal degradation product	106	C_8_H_10_	106-42-3	902	860	-	-	-	2.39 ± 0.22
7.40	Benzaldehyde	PET recycled indicator	106	C_7_H_6_O	100-52-7	966	970	-	-	-	0.36 ± 0.14
9.06	dl-Limonene	PET recycled indicator	136	C_10_H_16_	138-86-3	1025	1032	-	-	-	7.86 ± 0.68
14.16	Benzoic acid	PET thermal degradation product	150	C_9_H_10_O_2_	93-89-0	1177	1160	-	-	-	4.75 ± 0.83
30.13	Dodecanoic acid	Lubricant	200	C_12_H_24_O_2_	143-07-7	1500	1562	4.36 ± 0.33	-	6.19 ± 0.37	-
30.74	Diethyl phthalate	Plasticizer	222	C_12_H_14_O_4_	84-66-2	1571	1546	18.73 ± 5.14	7.32 ± 4.48	-	-
36.77	3,5-Di-tert-butyl-4-hydroxybenzaldehyde	BHT oxidation product	234	C_15_H_22_O_2_	1620-98-0	1701	1774	-	-	2.63 ± 1.50	-
37.40	Tetradecanoic acid	Lubricant	228	C_14_H_28_O_2_	544-63-8	1750	1761	6.87 ± 0.10	3.79 ± 0.50	5.64 ± 0.68	4.78 ± 1.50
40.84	Pentadecanoic acid	Lubricant	242	C_15_H_30_O_2_	1002-84-2	1842	1855	1.17 ± 0.87	-	-	-
44.35	Hexadecanoic acid	Lubricant	256	C_16_H_32_O_2_	57-10-3	1961	1957	52.74 ± 2.21	59.07 ± 7.71	68.16 ± 0.79	55.30 ± 3.70
50.36	Octadecanoic acid	Lubricant	284	C_18_H_36_O_2_	57-11-4	2147	2170	16.13 ± 4.41	29.83 ± 3.31	17.39 ± 1.98	24.56 ± 1.30

^a^ Values are the mean and standard deviation of three replicates. ^b^ Kovats index calculated in a DB5MS column. ^c^ From NIST (available online: http://webbook.nist.gov/chemistry/cas-ser.html, last accessed on 28 May 2021) for DB5 column; - means not present.

**Table 2:** GC-MS peak relative areas ^a^ of compounds identified in PET bottle samples.

Tr (min)	Compounds	Function/Origin	Mw	Formula	CAS	KIcal ^b^	KI Ref ^c^	P	T	A	D
5.64	p-Xylene	Thermal degradation product	106	C_8_H_10_	106-42-3	903	860	1.68 ± 0.69	-	-	1.69 ± 0.60
9.06	dl-Limonene	PET recycled indicator	136	C_10_H_16_	138-86-3	1026	1020	-	1.94 ± 0.14	-	1.08 ± 0.05
11.53	Nonanal	Thermal degradation product from PE waxes (lubricants)	142	C_9_H_18_O	124-19-6	1104	1071	2.14 ± 1.25	-	1.03 ± 0.51	0.41 ± 0.30
12.13	Triethyl phosphate	Plasticizer	182	C_6_H_15_O_4_P	78-40-0	1122	1120	10.06 ± 5.24	-	-	0.92 ± 0.19
14.16	Benzoic acid	PET thermal degradation product	122	C_7_H_6_O_2_	65-85-0	1172	1170	-	1.72 ± 0.69	-	-
15.41	Decanal	Thermal degradation product from PE waxes (lubricants)	156	C_10_H_20_O	112-31-2	1206	1214	-	-	-	0.26 ± 0.10
18.52	Nonanoic acid	Lubricant	158	C_9_H_18_O_2_	112-05-0	1270	1278	-	-	-	1.15 ± 0.21
25.76	2,6-Di-tert-butyl-1,4-benzoquinone	Irganox degradation product	212	C_14_H_20_O_2_	719-22-2	1449	1458	-	0.50 ± 0.12	-	0.46 ± 0.19
27.75	Phenol, 2,4-bis(1,1-dimethylethyl)-	Degradation product from phosphite-based antioxidant–process stabilizer	206	C_14_H_22_O	96-76-4	1495	1502	1.45 ± 1.12	0.43 ± 0.12	-	0.97 ± 0.14
30.13	Dodecanoic acid	Lubricant	200	C_12_H_24_O_2_	2305-05-7	1554	1562	3.36 ± 1.65	1.69 ± 0.21	5.96 ± 0.94	2.40 ± 1.52
30.74	Diethyl phthalate	Plasticizer	222	C_12_H_14_O_4_	84-66-2	1572	1546	2.65 ± 0.43	4.08 ± 0.92	24.74 ± 10.74	1.16 ± 0.61
31.34	Hexadecane	From paraffine wax (lubricant)	226	C_16_H_34_	544-76-3	1588	1603	0.77 ± 0.12	-	-	-
32.12	Benzophenone	PET recycled indicator	182	C_13_H_10_O	119-61-9	1608	1644	0.74 ± 0.15	0.54 ± 0.04	-	0.47 ± 0.16
37.40	Tetradecanoic acid	Lubricant	228	C_14_H_28_O_2_	544-63-8	1773	1756	4.88 ± 0.44	2.74 ± 0.47	-	4.98 ± 1.46
40.84	Pentadecanoic acid	Lubricant	242	C_15_H_30_O_2_	1002-84-2	1842	1855	-	-	-	4.70 ± 2.48
41.81	7,9-Di-tert-butyl-1-oxaspiro(4,5)deca-6,9-diene-2,8-dione	Possible degradation product from Irgafox 168	276	C_17_H_24_O_3_	82304-66-3	1873	1916	-	-	2.97 ± 0.39	-
44.35	Hexadecanoic acid	Lubricant	256	C_16_H_32_O_2_	57-10-3	1981	1972	44.54 ± 3.88	56.67 ± 0.87	46.59 ± 8.63	45.80 ± 8.08
47.22	Heptadecanoic acid	Lubricant	270	C_17_H_34_O_2_	506-1 2-7	2043	2080	-	2.49 ± 0.35	-	-
48.34	Heneicosane	From paraffine wax (lubricant)	296	C_21_H_44_	629-94-7	2088	2100	2.96 ± 0.95	1.47 ± 0.72	7.52 ± 2.05	-
50.36	Octadecanoic acid	Lubricant	284	C_18_H_36_O_2_	57-11-4	2144	2158	25.14 ± 2.11	26.36 ± 3.47	11.21 ± 6.21	33.54 ± 7.27

^a^ Values are the mean and standard deviation of three replicates. ^b^ Kovats index calculated in a DB5MS column. ^c^ From NIST (available online: http://webbook.nist.gov/chemistry/cas-ser.html, last accessed on 28 May 2021) for DB5 column; - means not present.

## Data Availability

Not applicable.

## Supplementary Materials

The following are available online at https://www.mdpi.com/article/10.3390/polym13213769/s1, Figure S1: Chromatograms of PET bottles A and D, Table S1: Unknown substances found in PET bottles P, Table S2: Unknown substances found in PET bottles T, Table S3: Unknown substances found in PET bottles A, Table S4: Unknown substances found in PET bottles D.
