# Supplementary material for: Identification of Potential Migrants in Polyethylene Terephthalate Samples of Ecuadorian Market
Source: Polymers (Basel). 2021 Oct 31;13(21):3769. doi: 10.3390/polym13213769 (PMC8588110; doi:10.3390/polym13213769)
Supplement: Supplementary file 1 [file polymers-13-03769-s001.zip › Supplementary information.pdf]

Supplementary information

Identification of potential migrants in Polyethylene terephthalate samples of Ecuadorian market

Karina Marín-Morocho [a], Sandra Domenek [b], Rómulo Salazar [a,*]

[a] Escuela Superior Politécnica del Litoral, ESPOL, Facultad de Ingeniería en Mecánica y Ciencias de la Producción, Carrera de Ingeniería en Alimentos, Campus Gustavo Galindo Km 30.5 Vía Perimetral, P.O. Box 09-01-5863, Guayaquil, Ecuador

[b] UMR Ingénierie Procédés Aliments, AgroParisTech, INRA, Université Paris-Saclay, F-91300, Massy, France.

* Corresponding author: rvsalaza@espol.edu.ec

Tables presenting the information gathered on the non-identified peaks present in the PET bottles sampled on the Ecuadorian market

Table S1: Unknown substances found in PET bottles P.

t (min)	Compounds	m/z ratio	KI[a]
10.42	unknown	117, 201, 119, 203	1066
40.40	unknown	149, 57, 150, 223	1836
41.97	unknown	149, 57, 71, 85	1877
42.75	unknown	74, 87, 55, 75	1905
43.53	unknown	149, 150, 223, 104	1927
45.20	unknown	57, 71, 85, 55, 99	1974
48.90	unknown	71, 57, 85, 69, 99	2096
51.23	unknown	57, 71, 85, 55, 99	2157
54.09	unknown	57, 71, 85, 55, 69	2257
56.82	unknown	57, 71, 85, 55, 69	2361
59.45	unknown	57, 71, 85, 55, 69	2462
59.71	unknown	55, 69, 83, 97, 57	2473
60.04	unknown	149, 167, 57, 71	2485

[a] Kovats index calculated in a DB5MS column.

Table S2: Unknown substances found in PET bottles T.

t (min)	Compounds	m/z ratio	KI[a]
10.45	unknown	201, 117, 119, 203	1066
18.43	unknown	91, 136, 119, 65	1311
40.40	unknown	149, 57, 150, 223	1836
40.80	unknown	57, 73, 171, 71	1846
42.80	unknown	74, 87, 57, 55	1906
43.56	unknown	149, 57, 150, 71	1928
51.09	unknown	88, 101, 57, 55	2154
54.14	unknown	57, 71, 85, 55	2260
56.86	unknown	57, 71, 85, 55	2362
60.08	unknown	149, 167, 57, 71	2489

[a] Kovats index calculated in a DB5MS column.

Table S3: Unknown substances found in PET bottles A.

t (min)	Compounds	m/z ratio	KI[a]
6.61	unknown	143, 145, 83, 147	931
10.42	unknown	201, 117, 119, 203	1066
12.37	unknown	96, 143, 145, 83	1121
14.48	unknown	128, 56, 127, 129	1174
37.30	unknown	57, 73, 60, 55, 71	1743
38.56	unknown	57, 71, 85, 55, 99	1784
40.39	unknown	149, 57, 150, 223	1835
41.94	unknown	149, 57, 71, 85	1877
42.74	unknown	74, 87, 55, 75	1905
43.51	unknown	149, 150, 223, 104	1927
45.18	unknown	57, 71, 85, 55	1974
51.23	unknown	57, 71, 85, 55	2157
54.04	unknown	57, 71, 85, 55	2255
56.78	unknown	57, 71, 85, 55	2359
59.99	unknown	149, 167, 57, 71	2485

[a] Kovats index calculated in a DB5MS column.

Table S4: Unknown substances found in PET bottles D.

t (min)	Compounds	m/z ratio	KI[a]
6.52	unknown	83, 85, 95, 131	926
6.71	unknown	79, 103, 75, 107	935
21.32	unknown	71, 83, 56, 98	1356
22.29	unknown	71, 89, 56, 73	1373
34.93	unknown	71, 57, 85, 99	1672
36.54	unknown	71, 57, 85, 99	1717
40.43	unknown	149, 57, 150, 223	1835
42.78	unknown	74, 87, 177, 195	1898
43.59	unknown	149, 150, 57, 223	1920
51.09	unknown	88, 101, 57, 55	2154
59.46	unknown	57, 71, 85, 55	2462
60.10	unknown	149, 167, 57, 71	2490

[a] Kovats index calculated in a DB5MS column.

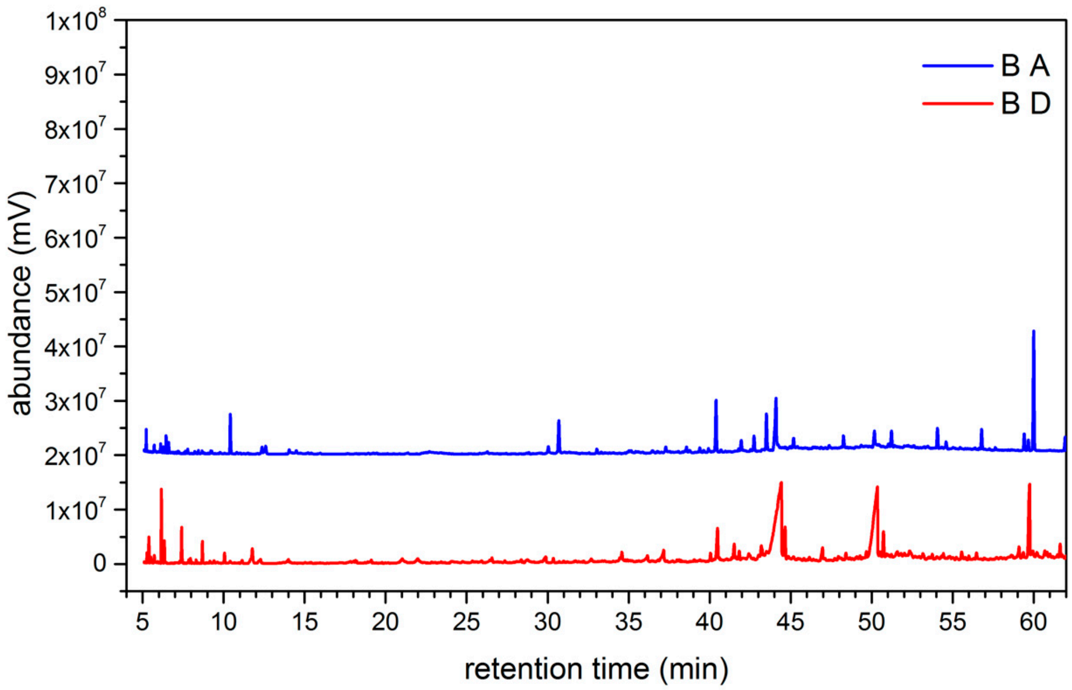

Figure S1: Chromatograms of PET bottles A and D.
